# Supplementary material for: Private-Public Opinion Discrepancy
Source: PLoS One. 2020 Nov 25;15(11):e0242148. doi: 10.1371/journal.pone.0242148 (PMC7688181; doi:10.1371/journal.pone.0242148)
Supplement: S1 File — (ZIP) [file pone.0242148.s001.zip › Private-Public attitude Discrepancy (Matlab Code).pdf]

```
%% Paper title: Private-Public attitude Discrepancy
%% Authors: Manfredi, R., Guazzini, A., Roos, C.A., Postmes, T., Koudenburg, N.
%% Reference code for numerical simulations
%% Model Control Parameters
% Agents species: C= Consensus Seekers, R= Reputation Seekers (i.e., Agents' type)
% Private Agents' attitudes: A or B (i.e., Agents' attitude)
%% Model Order Parameter
% Agents' Behaviour: Silence or Expression (i.e., Public behaviour)

close all
clear all

% System parameters
N=100;      % Number of agents
T_Max=1000;  % Number of interactions within 1 stat

% Network (Group) representation and agent Creation (C and R)
A=zeros(N,3); % Agents' state vector (Column 1- Agent's Type (0=C or 1=R); Column
2- Agent's attitude (1=A or 1=B); Column 3- Agent's Public Behaviour (0=Silence;
1=Expression)
Network_S=zeros(sqrt(N),sqrt(N)); % Agents' Type matrix creation
Network_O=zeros(sqrt(N),sqrt(N)); % Agents' Attitude matrix creation
Network_E=zeros(sqrt(N),sqrt(N)); % % Agents' Public Behaviour matrix creation
Old_Network_E=zeros(sqrt(N),sqrt(N)); % Matrix to manage the parallel updating of
the system

for i =1:N,
    A(i,1)=round(rand); % Random selection of agents' type (0=C and 1=R)
    A(i,2)=round(rand)+1; % Random selection of agents' attitude  (1=A and 2=B)
    A(i,3)=round(rand); % Random seeding of agents' initial public behaviour  (0=S
and 1=E)

    c=mod(i-1,sqrt(N))+1;   % Finding of the column where the agent i is located
    r=floor((i-1)/sqrt(N))+1; % Finding of the row where the agent i is located

    Network_S(r,c)=A(i,1); % Agents' type network
    Network_O(r,c)=A(i,2); % Agents' attitude network
    Network_E(r,c)=A(i,3); % Agents' public behaviour network
end


%% Main cycle exploring the effect of Reputation seekers density (percentage)

NR=0;       % Initial number of Reputation seekers (R) (Obviously NC=N-NR)
NC=N-NR;    % Number of Consensus seekers
Step_NR=N+1; % Number of Simulation steps
Stat=10000;   % Number of statistics

Results_S=zeros(N+1,Stat,14);   % Statistics aggregated Results: Col 1: Average
Silence Dynamics (T_max/2:T_max); 2: Average of Real Delta attitude; 3: Average of
Expressed Delta attitude;
                                % 4-8: Agents' perceived dyversity; 9: Average
standard deviation of majority; 10: Max cycle lenght;
                                % 11: Min of Silent Majority Factor (SMF); 12: Max
of SMF; 13: Average SMF; 14: NR
Result_S_Std=zeros(N+1,Stat,14); % Results_S Standard Deviation

Results=zeros(N+1,14);    % Final results: Col 1: Average Silence Dynamics
(T_max/2:T_max); 2: Average of Real Delta attitude; 3: Average of Expressed Delta
attitude;
```

```
                           % 4-8: Agents' perceived dyversity; 9: Average standard
deviation of majority; 10: Max cycle lenght;
                           % 11: Min of Silent Majority Factor (SMF); 12: Max of
SMF; 13: Average SMF; 14: NR
Results_Std=zeros(N+1,14); % Standard deviations of Final Results vectors

Result_Special_Theta=zeros(N+1,Stat); % Results for Private-Public attitude
Discrepancy Index


for NR=0:N,

    for S=1:Stat,

        %% System Dynamics
        Mean_Expressed_attitude=zeros(1,T_Max);
        Silences=zeros(1,T_Max);
        Majority=zeros(2,T_Max); % 1-Number of agent expressing A, 2-Number of
agents expressing B
        Real_Majority=zeros(2,T_Max);
        Majority_Perception=zeros(sqrt(N),sqrt(N)); % The perception of Majority
Consensus changes at each time step, for each agent
        Silent_Majority_Factor=zeros(1,T_Max); % Vector to save the Gap between
Real and Expressed attitude (Silent Majority Effect/Regime)
        Silence_details=zeros(2,T_Max); % Percentage of R and C silenced at each
time step

        % Observables for the experiment
        Delta_Real_attitude=zeros(1,T_Max); % Difference between attitude A and B
        Delta_Expressed_attitude=zeros(2,T_Max); % Difference between expressed
attitudes (A and B)
        Std_Majority=zeros(1,T_Max); % Standard deviation of one of the two
attitude density (only when we have only 2 different attitudes)
        Dist_Cicle_Periods=[];    % Vector of Cycle Period at Equilibrum


            % Diversity_vector Update
            Diversity_Vector=zeros(5,T_Max); % Vector where the perceived diversity
map is collapsed within 4 density functions

            % Creation of a new random network
            % Network (Group) representation and agent Creation (C and R)
            A=zeros(N,3); % State vector of agents (1-Agent Type (0=C or 1=R); 2-
Real attitude by A(0=A or 1=B); 3-Expressed attitude (0=Silence; 1-Expression)
            Network_S=zeros(sqrt(N),sqrt(N)); % Lattice network!! Subject Typology
            Network_O=zeros(sqrt(N),sqrt(N)); % Lattice network!! attitude
            Network_E=zeros(sqrt(N),sqrt(N)); % Lattice network!! Silence or
Expression

            for i =1:N,
                A(i,1)=0; % 0=C and 1=R - First we create only Consensus Agents
                A(i,2)=round(rand*0.8)+1; % 1=A and 2=B NB: The factor 0.8 is
introduced in order to have always a real majority (Of course it can be changed to
study different regimes)
                A(i,3)=round(rand); % 0=S and 1=E

                c=mod(i-1,sqrt(N))+1;   % We find here the column here the agent i
is located
                r=floor((i-1)/sqrt(N))+1; % We find here the row where the agent i
is located
```

```
                Network_S(r,c)=A(i,1); % Network representation of Agents' nature
                Network_O(r,c)=A(i,2); % Network representation of Agents' nature
                Network_E(r,c)=A(i,3); % Network representation of Agents' nature
            end

            % Random Reputationists Seeding
            i=NR;

            while i>0,

                j=floor(rand*N)+1;
                if A(j,1)==0, % If the extracted agent is a Consensus agent it is
flipped to a Reputationist
                    A(j,1)=1;

                    c=mod(j-1,sqrt(N))+1;    % We find here the column here the
agent i is located
                    r=floor((j-1)/sqrt(N))+1; % We find here the row where the
agent i is located

                    Network_S(r,c)=A(j,1); % Network representation of Agents' type
                    i=i-1;
                end
            end

            for t=1:T_Max, % Time of the epoch


Majority(1,t)=sum(sum((Network_E.*Network_O)==1))/sum(sum(Network_E));

Majority(2,t)=sum(sum((Network_E.*Network_O)==2))/sum(sum(Network_E));
                Real_Majority(1,t)=sum(sum(Network_O==1))/N;
                Real_Majority(2,t)=sum(sum(Network_O==2))/N;

Mean_Expressed_attitude(1,t)=(sum(sum(Network_O.*Network_E)))/sum(sum(Network_E));
                Silences(1,t)=N-(sum(sum(Network_E)));

                % Update Delta
                Delta_Real_attitude(1,t)=abs(Real_Majority(1,t)-
Real_Majority(2,t)); % Absolute value of the difference between the two clusters of
attitude (This is a costant if the initial conditions remain the same)
                Delta_Expressed_attitude(1,t)=abs(Majority(1,t)-Majority(2,t)); %
                if sign(Majority(1,t)-Majority(2,t)) == sign(Real_Majority(1,t)-
Real_Majority(2,t)), % If the Real Majority is also the Real Expressed Majority
                    Silent_Majority_Factor(1,t)=abs((abs(Majority(1,t)-
Majority(2,t))-abs(Real_Majority(1,t)-Real_Majority(2,t)))/abs(Real_Majority(1,t)-
Real_Majority(2,t)));
                elseif sign(Real_Majority(1,t)-Real_Majority(2,t))~=0;
                    Silent_Majority_Factor(1,t)=-abs((abs(Majority(1,t)-
Majority(2,t))-abs(Real_Majority(1,t)-Real_Majority(2,t)))/abs(Real_Majority(1,t)-
Real_Majority(2,t)));
                else % The sign of Real_Majority difference is Zero

                end

                if t>20
                Std_Majority(1,t)=std(Majority(1,t-20:t)); % We adopt a temporal
window of 10 in order to capture both limit cycles and fixed equilibria
                end
```

```
                % Update Perceived Dyversity
                Diversity_Vector(1,t)=sum(sum(Majority_Perception==0)); % Time line
of "Silences"
                Diversity_Vector(2,t)=sum(sum(Majority_Perception==1)); % Time line
of "Stalemate"
                Diversity_Vector(3,t)=sum(sum(Majority_Perception==2)); % Time line
of "Majority with Minority around"
                Diversity_Vector(4,t)=sum(sum(Majority_Perception==3)); % Time line
of "Partial Majority only around"
                Diversity_Vector(5,t)=sum(sum(Majority_Perception==4)); % Time line
of "Full Majority only around"


                % Each agent perceives the majority around him

                Old_Network_E=Network_E;    % The world is saved in order to update
the system in parallel
                Silences_R=[];    % Vector of R silenced
                Silences_C=[];    % Vector of C silenced

                for i=1:N,
                   % Finding the coordinates of agent i
                   c=mod(i-1,sqrt(N))+1;    % We find here the column where the
agent i is located
                   r=floor((i-1)/sqrt(N))+1; % We find here the row where the agent
i is located


                   % Agent i accumulates the attitude from its neighbours if they
expressed it
                   O_test=zeros(1,2);    % Temporary memory of agent i regarding the
attitudes of its neighbours
                   for nr=-1:2:1, % Explore the 2 row neighbours
                       cn=c+nr; % The column of my neighbour
                       if cn==0, cn=sqrt(N); end
                       if cn==sqrt(N)+1, cn=1; end

                       % Neighbour analysis of attitude
                       if Old_Network_E(r,cn)==1, % If the neigbour expressed its
attitude
                           attitude=Network_O(r,cn);  % I take the attitude of
agent i
                           O_test(1,attitude)=O_test(1,attitude)+1; % Updating of
O_test for agent i
                       end
                   end
                   for nc=-1:2:1, % Explore the 2 column neighbours
                       rn=r+nc; % The row of my neighbour
                       if rn==0, rn=sqrt(N); end
                       if rn==sqrt(N)+1, rn=1; end

                       % Neighbour analysis of attitude
                       if Old_Network_E(rn,c)==1, % If the neigbour expressed its
attitude
                           attitude=Network_O(rn,c);  % I take the attitude of
agent i
                           O_test(1,attitude)=O_test(1,attitude)+1; % Updating of
O_test for agent i
                       end
```

```
                end



                % The agent Decides if express (I.e., modify the Network_E) its
attitude or not
                %% Equations of OUR model (Manfredi-Postmes-Guazzini-Roos-
Koudenburg)
                if abs(O_test(1,1)-O_test(1,2))>0, % There is a Majority
                    [V,Dominant_attitude]=max(O_test);

                       if Network_O(r,c)==Dominant_attitude && min(O_test)==0,
% If the Agent's attitude is equal to the perceived Majority and there is no
Minority
                          if Network_S(r,c)==0, % If the agent is a C agent
(Consensus seeker)
                             Network_E(r,c)=1;   % The agent i expresses its
opinion!
                          elseif Network_S(r,c)==1, % If the agent is a R
agent (Reputation seeker)
                             Network_E(r,c)=0;   % The agent i shut-up
(Silence)
                          end
                       elseif Network_O(r,c)==Dominant_attitude &&
min(O_test)>0, % If the Agent's attitude is equal to the perceived Majority and
there is a Minority
                          if Network_S(r,c)==0, % If the agent is a C agent
(Consensus seeker)
                             Network_E(r,c)=0;   % The agent shut up!!
(Silence)
                          elseif Network_S(r,c)==1, % If the agent is a R
agent (Reputation seeker)
                             Network_E(r,c)=0;   % The agent i shut-up
(Silence)
                          end
                       else % If the attitude of i is different from the
majority
                          if Network_S(r,c)==0 && min(O_test)>0, % If the
agent is a C agent (Consensus seeker) and someone else expressed the minority
attitude
                             Network_E(r,c)=0;   % The agent i shut-up
(Silence)
                          elseif Network_S(r,c)==0 && min(O_test)==0, % If the
agent is a C agent (Consensus seeker) and noone else expressed the minority
attitude
                             Network_E(r,c)=0;   % The agent i shuts-up
(Silence)
                          elseif Network_S(r,c)==1 && min(O_test)>0, % If the
agent is a R agent (Reputation seeker) and someone else expressed the minority
attitude
                             Network_E(r,c)=1;   % The agent i speaks
                          elseif Network_S(r,c)==1 && min(O_test)==0, % If the
agent is a R agent (Reputation seeker) and noone else expressed the minority
attitude
                             Network_E(r,c)=0;   % The agent i shuts-up
(Silence)
```

```
                    end
                end
            elseif O_test(1,1)==O_test(1,2) && O_test(1,1)>0, % STALEMATE
There is no a majority without complete silence
                if Network_S(r,c)==0, % If the agent is a C agent (Consensus
seeker)
                    Network_E(r,c)=0;   % The agent i shuts-up (Silence)
                elseif Network_S(r,c)==1, % If the agent is a R agent
(Reputation seeker)
                    Network_E(r,c)=1;   % The agent i expressed its attitude
                end
            elseif O_test(1,1)==O_test(1,2) && O_test(1,1)==0, % Complete
Silence
                if Network_S(r,c)==0, % If the agent is a C agent (Consensus
seeker)
                    Network_E(r,c)=0;   % The agent i shuts-up (Silence)
                elseif Network_S(r,c)==1, % If the agent is a R agent
(Reputation seeker)
                    Network_E(r,c)=1;   % The agent i expressed its attitude
                end
            end

            % Recording of silences divided for R and C
            if Network_S(r,c)==1 && Network_E(r,c)==0, % (Reputation Seekers
Silenced)
                Silences_R=[Silences_R 1];    % Vector of R silences
            elseif Network_S(r,c)==1 && Network_E(r,c)==1, % (Reputation
Seekers Expressing)
                Silences_R=[Silences_R 0];    % Vector of R silences
            elseif Network_S(r,c)==0 && Network_E(r,c)==0, % (Consensus
Seekers Silenced)
                Silences_C=[Silences_C 1];    % Vector of C silences
            elseif Network_S(r,c)==0 && Network_E(r,c)==1, % (Consensus
Seekers Expressing)
                Silences_C=[Silences_C 0];    % Vector of C silences
            end

            %  Updating the Perceived Difference of its neighboroud
            if max(O_test)==0, % No one expressed its attitude around i
                 Majority_Perception(r,c)=0;
            elseif min(O_test)>0 && min(O_test)==max(O_test), % Perfect
symmetry of expressed attitudes, no majority (Stelemate)
                 Majority_Perception(r,c)=1;
            elseif min(O_test)>0 && min(O_test)<max(O_test), % There is a
majority as well as a minority expressed around i
                 Majority_Perception(r,c)=2;
            elseif min(O_test)==0 && max(O_test)>0 && max(O_test)<4, % There
is only a majority around i with silence minority
                 Majority_Perception(r,c)=3;
            elseif max(O_test)==4, % There is only a majority around i and
no silenced agents
                 Majority_Perception(r,c)=4;
            end


        end % N cycle

        % Saving the silences_R and C percentage
        Silence_details(1,t)=(sum(Silences_R)/length(Silences_R))*100; %
```

```
Percentage of R agents silenced
                Silence_details(2,t)=(sum(Silences_C)/length(Silences_C))*100; %
Percentage of C agents silenced

                % Majority
                 % Calculation of the Cycle Period by means of evaluation of the
past experience of the system (i.e., expressed attitude)
                 if t>20
                 for i=t-1:-1:1,
                     if Majority(1,t)==Majority(1,i);
                         Dist_Cicle_Periods=[Dist_Cicle_Periods t-i]; % The
estimated lenght of period is t-i
                         break % In this case the cycle is stopped
                     end
                 end
                 end




            end % Epoch end

        %% Results updating

        Results_S(NR+1,S,1)=mean(Silences(1,T_Max/2:T_Max)); % Average value of
silence dynamics
        Results_S(NR+1,S,2)=mean(Delta_Real_attitude(1,T_Max/2:T_Max)); % Average
real Delta between attitudes (Percentage)
        Results_S(NR+1,S,3)=mean(Delta_Expressed_attitude(1,T_Max/2:T_Max)); %
Average expressed Delta between attitudes (Percentage)
        Results_S(NR+1,S,4)=mean(Diversity_Vector(1,T_Max/2:T_Max)); % Average
number of Total Silence
        Results_S(NR+1,S,5)=mean(Diversity_Vector(2,T_Max/2:T_Max)); % Average
number of Stalemate
        Results_S(NR+1,S,6)=mean(Diversity_Vector(3,T_Max/2:T_Max)); % Average
number of Majority with Minority
        Results_S(NR+1,S,7)=mean(Diversity_Vector(4,T_Max/2:T_Max)); % Average
number of Partial Majority with Silence
        Results_S(NR+1,S,8)=mean(Diversity_Vector(5,T_Max/2:T_Max)); % Average
number of Full Majority with no Silence
        Results_S(NR+1,S,9)=mean(Std_Majority(1,T_Max/2:T_Max)); % Average standard
deviation of majority
        [a,b]=size(Dist_Cicle_Periods);
        if a==0 && b==0, Dist_Cicle_Periods=T_Max; end
        Results_S(NR+1,S,10)=max(Dist_Cicle_Periods(1,:)); % Max Cycle Length
        Results_S(NR+1,S,11)=min(Silent_Majority_Factor(1,T_Max/2:T_Max)); % Min of
Private-Public attitude Discrepancy Index
        Results_S(NR+1,S,12)=max(Silent_Majority_Factor(1,T_Max/2:T_Max)); % Max of
Private-Public attitude Discrepancy Index
        Results_S(NR+1,S,13)=mean(Silent_Majority_Factor(1,T_Max/2:T_Max)); %
Average Private-Public attitude Discrepancy Index
        Results_S(NR+1,S,14)=NR+1; % Number of reputationist
        Results_S(NR+1,S,15)=mean(Silence_details(1,T_Max/2:T_Max));  % Percentage
of silenced R agents
        Results_S(NR+1,S,16)=mean(Silence_details(2,T_Max/2:T_Max));  % Percentage
of silenced C agents


Results_Special_Theta(NR+1,S)=mean(Silent_Majority_Factor(1,T_Max/2:T_Max)); %
Average of Private-Public attitude Discrepancy Index
```

```
        % Saving of Min, Max and STD of the previous dimensions
        Results_S_Std(NR+1,S,1)=std(Silences(1,T_Max/2:T_Max)); % Std of value of
silence dynamics
        Results_S_Std(NR+1,S,2)=std(Delta_Real_attitude(1,T_Max/2:T_Max)); % Std of
real Delta between attitudes (Percentage)
        Results_S_Std(NR+1,S,3)=std(Delta_Expressed_attitude(1,T_Max/2:T_Max)); %
Std of expressed Delta between attitudes (Percentage)
        Results_S_Std(NR+1,S,4)=std(Diversity_Vector(1,T_Max/2:T_Max)); % Std of
number of Total Silence
        Results_S_Std(NR+1,S,5)=std(Diversity_Vector(2,T_Max/2:T_Max)); % Std of
number of Stalemate
        Results_S_Std(NR+1,S,6)=std(Diversity_Vector(3,T_Max/2:T_Max)); % Std of
number of Majority with Minority
        Results_S_Std(NR+1,S,7)=std(Diversity_Vector(4,T_Max/2:T_Max)); % Std of
number of Partial Majority with Silence
        Results_S_Std(NR+1,S,8)=std(Diversity_Vector(5,T_Max/2:T_Max)); % Std of
number of Full Majority with no Silence
        Results_S_Std(NR+1,S,9)=std(Std_Majority(1,T_Max/2:T_Max)); % Std of
standard deviation of majority
        Results_S_Std(NR+1,S,10)=max(Dist_Cicle_Periods(1,:)); % Max Cycle Length
        Results_S_Std(NR+1,S,11)=min(Silent_Majority_Factor(1,T_Max/2:T_Max)); %
Min of Private-Public attitude Discrepancy Index
        Results_S_Std(NR+1,S,12)=max(Silent_Majority_Factor(1,T_Max/2:T_Max)); %
Max of Private-Public attitude Discrepancy Index
        Results_S_Std(NR+1,S,13)=std(Silent_Majority_Factor(1,T_Max/2:T_Max)); %
Std of Private-Public attitude Discrepancy Index
        Results_S_Std(NR+1,S,14)=NR+1; % Number of reputationist
        Results_S_Std(NR+1,S,15)=std(Silence_details(1,T_Max/2:T_Max));  % Std of
silenced R agents
        Results_S_Std(NR+1,S,16)=std(Silence_details(2,T_Max/2:T_Max));  % Std of
silenced C agents
    end % Statistics

        % Here we mediate on the statistics
        Results(NR+1,1)=nanmean(Results_S(NR+1,:,1)); % Average value of silence
dynamics statistics
        Results(NR+1,2)=nanmean(Results_S(NR+1,:,2)); % Average real Delta between
attitudes statistics (Percentage)
        Results(NR+1,3)=nanmean(Results_S(NR+1,:,3)); % Average expressed Delta
between attitudes statistics (Percentage)
        Results(NR+1,4)=nanmean(Results_S(NR+1,:,4)); % Average number of Total
Silence statistics
        Results(NR+1,5)=nanmean(Results_S(NR+1,:,5)); % Average number of Stalemate
statistics
        Results(NR+1,6)=nanmean(Results_S(NR+1,:,6)); % Average number of Majority
with Minority statistics
        Results(NR+1,7)=nanmean(Results_S(NR+1,:,7)); % Average number of Partial
Majority with Silence statistics
        Results(NR+1,8)=nanmean(Results_S(NR+1,:,8)); % Average number of Full
Majority with no Silence statistics
        Results(NR+1,9)=nanmean(Results_S(NR+1,:,9)); % Average standard deviation
of majority statistics
        Results(NR+1,10)=nanmean(Results_S(NR+1,:,10)); % Max Cycle Length
statistics
        Results(NR+1,11)=nanmean(Results_S(NR+1,:,11)); % Min of Private-Public
attitude Discrepancy Index statistics
        Results(NR+1,12)=nanmean(Results_S(NR+1,:,12)); % Max of Private-Public
attitude Discrepancy Index statistics
        Results(NR+1,13)=nanmean(Results_S(NR+1,:,13)); % Average Private-Public
```

```
attitude Discrepancy Index statistics
        Results(NR+1,14)=nanmean(Results_S(NR+1,:,14)); % Number of reputationist
        Results(NR+1,15)=nanmean(Results_S(NR+1,:,15)); % Percentage of silenced R
agents statistics
        Results(NR+1,16)=nanmean(Results_S(NR+1,:,16)); % Percentage of silenced C
agents statistics

        % Here we mediate on the statistics for STD
        Results_Std(NR+1,1)=nanmean(Results_S_Std(NR+1,:,1)); % Average value of
STD silence dynamics
        Results_Std(NR+1,2)=nanmean(Results_S_Std(NR+1,:,2)); % Average real STD
Delta between attitudes (Percentage)
        Results_Std(NR+1,3)=nanmean(Results_S_Std(NR+1,:,3)); % Average expressed
STD Delta between attitudes (Percentage)
        Results_Std(NR+1,4)=nanmean(Results_S_Std(NR+1,:,4)); % Average STD of
number of Total Silence
        Results_Std(NR+1,5)=nanmean(Results_S_Std(NR+1,:,5)); % Average STD of
number of Stalemate
        Results_Std(NR+1,6)=nanmean(Results_S_Std(NR+1,:,6)); % Average STD of
number of Majority with Minority
        Results_Std(NR+1,7)=nanmean(Results_S_Std(NR+1,:,7)); % Average STD of
number of Partial Majority with Silence
        Results_Std(NR+1,8)=nanmean(Results_S_Std(NR+1,:,8)); % Average STD of
number of Full Majority with no Silence
        Results_Std(NR+1,9)=nanmean(Results_S_Std(NR+1,:,9)); % Average STD of
standard deviation of majority
        Results_Std(NR+1,10)=nanstd(Results_S_Std(NR+1,:,10)); % STD of Max Cycle
Length
        Results_Std(NR+1,11)=nanstd(Results_S_Std(NR+1,:,11)); % STD Min of
Private-Public attitude Discrepancy Index
        Results_Std(NR+1,12)=nanstd(Results_S_Std(NR+1,:,12)); % SYD Max of
Private-Public attitude Discrepancy Index
        Results_Std(NR+1,13)=nanmean(Results_S_Std(NR+1,:,13)); % Average STD
Private-Public attitude Discrepancy Index
        Results_Std(NR+1,14)=nanstd(Results_S_Std(NR+1,:,14)); % STD of Number of
reputationist
        Results_Std(NR+1,15)=nanmean(Results_S_Std(NR+1,:,15)); % STD Percentage of
silenced R agents
        Results_Std(NR+1,16)=nanmean(Results_S_Std(NR+1,:,16)); % STD Percentage of
silenced C agents

end % NR Cycle
```
